# Monthly Review of Parisian Medicine and Surgery

**Published:** 1859-07

**Authors:** Theophilus Mack

**Affiliations:** Lecturer on Materia Medica and Therapeutics in the Medical Department of the University of Buffalo


ART. III.—*Monthly Review of Parisian Medicine and Surgery.* By THEOPHILUS MACK, M. D., Lecturer on Materia Medica and Therapeutics in the Medical Department of the University of Buffalo.

The Germans, under the title of "*Gynecology*," have recognized a branch of medical science, embracing the description and treatment of all the maladies peculiar to woman. Notwithstanding the great attention of late years bestowed upon the disorders of the genital apparatus in the female, a complete treatise upon all those diseases, surgical as well as medical, internal and external, has only just been supplied by Scanzoni.

He divides his subject into seven parts: 1st. Of the Uterus; 2d. The Ligaments of the Uterus; 3d. Fallopian Tubes; 4th. The Ovaries; 5th. Vagina; 6th. Of the Vulva; 7th. Of the Breasts.

This work will furnish to the student and practitioner a ready reference upon diseases of the female sexual organs, without having recourse to several different authorities for information which here may be found in one volume. The author is an energetic advocate of sanguine emissions directly from the uterine neck. He expresses some doubts as to the importance attributed to the flexions of this organ, and condemns intra-uterine pessaries.

Dr. Fano reports a case of encephaloid cancer of the vaginal portion of the neck of the womb, cured by amputation. The part was pulled down and held by two pair of museux forceps until division of the diseased from the sound portion was effected by a long pair of curved scissors. The perchloride of iron arrested promptly all hemorrhage. The success of the operation has been complete.

A case of metrorrhagia dependent upon fungosities within the uterus, was treated successfully by digitaline, after failure with abrasion and cauterization. (*Gaz. des Hôpitaux.*)

Professor Gaillard, of Poitiers, has published a case of prolapsus uteri in a girl, ætat. 18 years, the result of a fall into a cellar; the hymen remaining unruptured. After a year of ineffectual treatment, the actual cautery was applied to the posterior part of the vagina, and by repeatedly performing this operation, the vagina was finally sufficiently narrowed to offer a resistance to the womb and prevent its descent.

During the discussion evoked at the Academy of Medicine, by the Memoir of M. Huguier, referred to in our last "*Review*," M. Dépaul stated catheterism of the uterus to be always dangerous. This opinion appears to us somewhat in rear of the results of experience. We have employed catheterism of this kind very frequently; and it has never been followed by any inconvenience, especially if the patient remained recumbent for a few hours after the operation. The most usual disagreeable effect of this maneuvre, is a peculiar sensation which "*goes to the heart*," and which vanishes after a few minutes.

Retroversio uteri, during the first moiety of the period of gestation, may be easily reduced, according to M. Godefroy, thus:

"The patient, sustained by assistants, is placed upon the front of her bed, the head and the hands supported upon the floor. The anterior part of the thighs and the legs alone repose upon the bed."

Two or three fingers introduced into the rectum easily then effect the desired reduction.

At the *Séance* of 30th March of the Chirurgical Society, M. Verneuil communicated an interesting observation of fracture of the leg, followed by aneurism.

Twenty hours after the accident, the leg was considerably tumefied, the skin tense and shining, the swelling hard and resisting, extending above the knee and almost doubling the volume of the foot. A dark blue color was perceived at certain points through the integument. Crepitation detected easily at the junction of the middle with the inferior third of the tibia, with tendency to displacement antero-posteriorly. The swelling concealed the fractured fibula; however, it was revealed by the abnormal mobility of all the inferior segment of the limb.

The phenomena which particularly attracted attention were, the existence of pulsations isochronous with those of the heart, and accompanied by expansion quite appreciable to the touch and even to the eye, seated at the antero-external region of the leg, between the tibia and fibula, at the seat of the fracture, and extending vertically about three inches. Compression of the femoral caused the phenomena to disappear.

The fracture having been reduced and properly treated, after the lapse of twenty days, the beatings and expansions at the point indicated were stronger than ever.

The patient, during a large portion of both day and night, from this time, exercised *digital compression;* in this way the current of blood was interrupted, on an average, for about eight hours in the twenty-four. After ten days of this treatment, there was manifest improvement; and the cure was completed by maintaining upon the course of the femoral a piece of lead, weighing about two pounds, inclosed in a bag.

A discussion took place at the next *séance* upon the subject of digital compression in aneurism; in which the value of this mode of treatment was fairly recognized.

M. Chassaignac presented two remarkable pathological specimens, viz., cancer of the gall-bladder, without any cancerous alteration of the liver; and fracture of the base of the skull, death occurring after two months, apparently from suppuration of the knee-joint, which resulted from direct injury sustained at the same time.

On the 27th April, M. Demarquay reported an instance of cancerous tumor of the lower part of the femur, in a child only seven years of age. Amputation was successfully performed, after first securing the femoral artery and vein.

M. Richet removed a hypertrophic tumor of the glandules of the lachrymal sac. This fact, which has no anterior case in the annals of science, completes the studies upon hypertrophic tumors of the mucous glandules in general, especially of the pituitary glands and their prolongations. Glandular hypertrophies of the lachrymal sac must henceforth be taken into consideration, in the differential diagnosis of tumors which produce themselves in the greater angle of the eye, and may give origin to *exorbitis.*

A review of the cases of traumatic fracture of the larynx hitherto reported, is called forth in the "*Gaz. des Hôpitaux*," by a fact at the "*Hotel Dieu*," of multiple fracture of the cartilages of the larynx, followed by emphysema and death. "The diagnosis of fractures of the larynx is founded upon the crepitus and abnormal mobility. The ecchymoses, swelling, dyspnœa and pain, may be the effect of a simple contusion. Crepitation may sometimes be absent—for instance, when the fracture is not complete. The mobility is then in reality the only truly pathognomonic sign."

At the Municipal Infirmary there is an unfortunate woman who presents 132 tumors of different volume upon her body, of a cancerous nature.

Professor Michel has cured a case of facial neuralgia, by section of the infra-orbital, inferior dental, buccal, and lingual nerves; about as heroic an exploit in neurotomy as we hear of in these "piping times" of surgical peace.

The "*Gazette Médicale de Lyon*" publishes the following case: An inhabitant of Brazil had a bull cut, and the animal died tetanic, probably from some defect in the mode of operating. He ordered the bull to be buried, but his slaves ate the meat by stealth. One of them was immediately seized with tetanus, and died in a short time. Two days afterward another died of the same affection, in the hospital; and a third was also admitted, suffering in the same manner, but was likely to recover.

M. Jobert de Lamballe, at the *Académie des Sciences*, presented a girl aged fourteen, affected with spasmodic contractions of the peronœus brevis, causing each time a loud noise. She was treated by subcutaneous section with success. This case was an example how, under the influence of muscular contraction, the displaced tendons can, at the instant of return into their osseous grooves, give rise to strokes resembling those which announce to certain persons the presence of the *knocking spirits*. M. Ch. Robin has communicated to the *Gaz. des Hôpitaux* a note upon this subject, from the pen of Prof. Flint, of Buffalo.

M. Ollier, having at a previous meeting communicated to the Academy a very interesting memoir upon the phenomena of the reproduction of bone by the periosteum, on the 28th March submitted a new work; in which he exposes the curious results of new experimental researches, whereby he proposes to follow the effects of the transplantation of bone, and of portions of periosteum, of one animal species to another.

The pathology of the eye is upon a new road to improvement, furnished by the ophthalmoscope and photography,—the first in permitting the direct examination of the deep membranes of the eye; the second, which had already rendered so many services to natural history and to pathological anatomy, in fixing the images, often transient, of the alterations in those membranes which rapidly undergo decomposition.

M. Labourdette has sent into the Academy of Medicine a work relative to the introduction of medicaments into milk, by digestive assimilation. He administers, with certain precautions, to the animals furnishing the milk, balls medicated with chlorides, arsenic, iodine, mercury, &c.; and thus proposes a very plausible mode of introducing these powerful agents into the delicate organism of early life.

In the *Journal de Médecine et de Chirurgie Pratique*, M. Bouchut reports three cases of croup evidently cured by large and repeated doses of tartar emetic. To a girl aged three years, he gave ten grains, in doses of two and a half grains every half-hour, in syrup of poppies. The cauterization of the larynx he recommends to be practiced at the same time, and the emetic given every half-hour, so that the child may throw up much, and not be too severely purged.

When the redness has gone after scarlatina and rubeola, the sequelæ are obviated at Metz by frictions with warm sweet-oil. A warm bath for one hour is given the next day; and two or three hours after, another friction is made, similar to the first.

M. Kuhn has recently published in the *Gaz. Médicale de Paris*, three cases of *cerebral rheumatism*. There existed in all the cases, from the commencement, in the midst of other rheumatic symptoms, pain, extending from the præcordial region to the left flank. There was an absence of fever, at first, and of all functional disturbance of the heart; and the febrile reaction was not manifested until after the invasion of the nervous centers. The two who died were deeply cyanosed; the one who recovered was so only in a minor degree. Lastly, the elevation of temperature, and the painfulness of the occipito-cervical region, with contraction of the muscles of the neck, were present in each of the three patients.

This form of rheumatism is met with as well in those who have not as who have taken quinine. It is a most serious form of the disease, terminating almost invariably by death, and remarkable by the absence of traces of inflammation, or lesion of any kind in the brain.

M. Henriette (*Jour. de Méd. de Bruxelles*) has succeeded in relieving children dying from exhausting diseases, by throwing decoction of bark and stimulants into the nostrils, by means of a small syringe. The fluids were readily swallowed, and two children were thus saved. This expedient furnishes a channel of election, when considered with the rectum, as avenues for the introduction of medicine or nourishment when the mouth is unavailable, and may in appropriate cases be deserving of consideration by the practitioner.

